# Metabolite profile of African horned cucumber (*Cucumis metuliferus* E. May. Ex Naudin) fruit grown under differing environmental conditions

**DOI:** 10.1038/s41598-022-07769-1

**Published:** 2022-03-08

**Authors:** Mdungazi Knox. Maluleke

**Affiliations:** Department of Environmental Sciences, College of Agriculture and Environmental Sciences, University of South Africa, Tshwane, South Africa

**Keywords:** Plant sciences, Environmental sciences

## Abstract

Plant metabolites are known as biological compounds that are essential to the growth and development of a plant and have a direct impact on yield and biochemical constituents of plants. For this study, the objective was to conduct primary metabolomics analysis using liquid chromatography mass spectrometry. African horned cucumber fruits were harvested from plants grown under pots experiment (greenhouse, shade net and open field), soil types (loamy soil and sandy loam) and three water stress levels (no water stress-100%—3L, moderate water stress-75%—2L, and severe water stress-35%—1L) during 2017/18 and 2018/19 seasons. Results showed that the treatment of no water stress combined with sandy loam under shade net environment, significantly increased asparagine content from 10 × 10^6^ to 80 × 10^6^ peak intensity. The treatment of no water stress, in combination with sandy loam soil under open field environment increased 4-hydroxyproline from 10 × 10^6^ to 90 × 10^6^ peak intensity compared to other treatments. It can be deduced that the treatment combination of (no water stress and moderate water stress) and all soil types, under greenhouse environment increased most metabolites content of the fruit when compared to other treatments. Therefore, it subsequently has potential to affect fruit quality such as taste and other biochemical constituents.

## Introduction

The endogenous metabolites, which happen to be of low molecular mass, provide a precise snapshot of the physiological state in biological samples^1–4^. A metabolite analysis of the fruit constituents would thus provide researchers with a molecular understanding of the response of the crop to the imposition of the varying treatments. The field of metabolomics is an emerging area that is mainly aimed at profiling the molecules present in plant materials such as stem, leaves and fruit^5^. The condition at which the plant was cultivated is likely to have positive or negative effects on the types of metabolites found in plant material^6^. Exploring the whole metabolomic profile provides a fuller, comprehensive understanding of biochemical constituents in plant materials than just focusing on a few molecules^7^. Osuji et al.^8^ observed the effect of temperature on primary metabolites profile of melon fruit and concluded that primary metabolites significantly increased in fruit harvested under a high temperature. This study looked at the effect of differing irrigation water levels and soil types under different growing conditions on the metabolites profile of African horned cucumber fruit. African horned cucumber is one of the most important indigenous crops in the Central and Southern Africa regions; however this crop is under-utilized, due to lack of knowledge on its importance and uses as an agronomic crop^9^. Osuji et al.^10^ indicated that only non-bitter varieties are palatable and have a similar blended taste of a mixture of banana and pineapple. The internal like-jelly content of African horned cucumber fruit can be scooped out and consumed as salad^11^.

The benefits of using the *C. metuliferus* fruits have been described and explained by several authors such as^12,13^ as being a good source of vitamin B, helping to rehydrate the body and replenishes daily vitamins, particularly A, for boosting vision, the fruit have a higher water content, which is essential in eliminating body toxins and low calorie that is ideal diet for people who are aiming to lose weight^14^. Finally,^15^, reported that the fruit also has medicinal properties that has a role in human metabolism.^12,16^ claimed that the fruit contains bioactive compounds that cure ulcers and are involved in regulation of blood pressure. With limited information on plant performance, most farmers may not grow the crop, because it may not be worth investing in it due to fear of risk of losses^17^. Providing scientifically proven information on the crop may, however, increase farmers' interest in adopting it for agronomic purposes. Variation in water levels are reported to have negative or positive effect on the primary metabolites of fruits by other researchers such as^4,8^. It is therefore evident that, at different water stress levels, there is a either a decrease or increase in metabolites content of fruit crops. This is due to the fact that under water stress, plant transpiration rate is reduced and subsequently cause imbalance in active osmoregulation of the plant^18^. In addition, it is often suggested that water stress has an effect on the metabolites content of fruit^19^. Kassu et al.^20^ also note that fruits harvested under a water stress environment tend to have higher metabolites content than those harvested from plants subjected to normal watering. They remarked that the maintenance of plant turgor and positive osmotic pressure from the canopy directly depends on the water absorption by roots. Singh et al. ^21^ concur with^22^, that fruit harvested from water stress conditions has high metabolites content due to the abnormal rate of enzymic activities caused by imbalanced liquid concentration within the xylem. Metabolite such as asparagine has been reported by^5^ as being responsible for filling of nitrogen in fruit seeds. However, stomatal closer due to water stress tend to negatively affect its concentration in fruits, since there is minimal gaseous exchange on the plant leaves.^23^ stated that metabolite dopa is known to be a compound released by plants, primarily to inhibit neighbouring plants' growth and dominance. Authors such as^4^ explained the function of metabolite niacinamide as being responsible for protection of cell leakage and DNA damage caused by environmental stress. These normally happens when there is competition for factors such water stress and space. It is well established that all aspects of plant growth, development and yield is affected by surrounding environmental conditions^24^. Metabolites profile in fruit will vary according to the conditions under which the plant is cultivated^25^. None of the studies linked metabolomics analysis of African horned cucumber fruit to varying environmental conditions. An understanding of how different water levels and soil types under varying growth environments will enable optimisation of management procedures and development of African horned cucumber crop cultivars that can tolerate adverse conditions and improve fruit quality. The objectives of this research were to assess differences between the profile of samples from the experimental treatments and quantify the different metabolites between samples so that breeders can have a better understanding of the actual cause of variation in fruit quality of African horned cucumber crop.

## Materials and methods

This study was conducted during [2017/18 and 2018/19] in a (greenhouse-controlled average temperature 16–28 °C, shade net-not controlled average 15–27 °C and open space environment-not controlled average 16–30 °C) at the Florida science campus of the University of South Africa (26° 10′ 30ʺ S, 27° 55′ 22.8ʺ E). Soil analysis was done at the Agricultural Research Council, Institute for Soil, Climate and Water (ARC-ISWC) in Pretoria (25° 44′ 19.4″ S 28° 12′ 26.4″ E). Sterilized growth media (loamy soil and sandy loam) were used. In addition, certified seeds of African horned cucumber were purchased from Seeds for Africa, Cape Town. A factorial experiment with two factors: soil (loamy soil and sandy loam soil) and three water stress levels (no water stress-100%—3L, moderate water stress-75%—2L, and severe water stress-35%—1L) during the 2017/18 and 2018/19irrigation water levels was determined. The pot experiment was a completely randomised design with nine (9) replicates per treatment and the factorial design as indicated above. The pots were spaced 1 m apart, and an up-rope vertical trellising was used to support the plants. On each site, plant pots were either filled with loamy soil or sandy loam. Each block comprised 18 plants in pots, resulting in 54 plants per site. A total of 162 plants were used for the experiment. Plants were well irrigated prior to imposition of the treatments. Irrigation water level (treatments) was imposed four weeks after seedlings establishment. It is worth to note that the experiment was conducted with strict adherence to UNISA, College of agriculture and Environmental Science Research and Higher degree committee.

### Physiological factors affecting *C. metuliferus* growth and development

#### Stomatal conductance

Chlorophyll content (µmol m^2^) (Stomatal conductance (mmol m^−2^ s^−1^) was measured at different growth stages (pre-flowering, flowering and fruiting) during the experimental period. The abaxial or lower leaf surface was measured, due to the fact that stomatal opening and conductance activities are more dominant on the lower leaf surface when compared to the upper surface (Savvides et al., 2012). The porometer (Delta-T Device, AP4 Leaf Porometer, United Kingdom) was used for the measurement of stomatal conductance, whereas (OPTI-SCIENCES-CCM 200 PLUS, USA) was used for chlorophyll content measurement.

#### Fresh fruits weight

**Figure 1:**
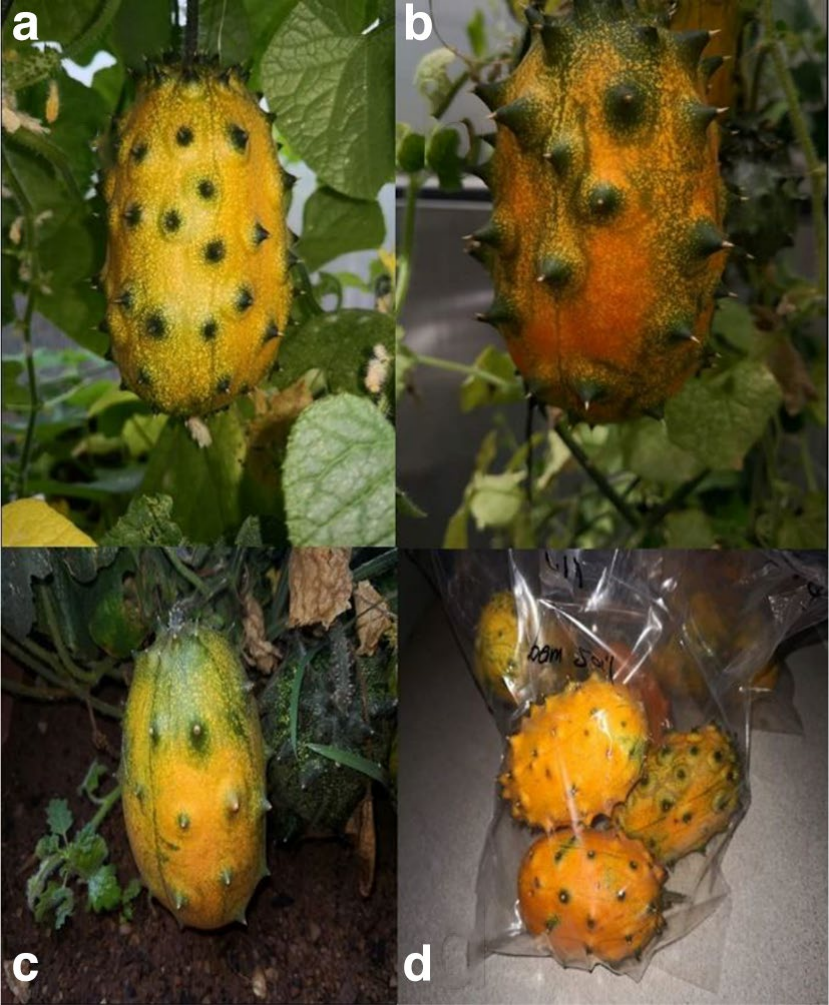
African horned cucumber fruit; (**a**) means fruit grown under (no water stress treatment); (**b**) means fruit grown under (moderate water stress treatment); (**c**) means fruit grown under (severe water stress treatment) and (**d**) means fully ripe fruits harvested for primary metabolites analysis.

Fresh ripe fruits (Fig. 1d) were harvested from plants cultivated in various soil types (loamy soil and sandy loam) and irrigation water levels (no water stress, moderate water stress, and severe water stress—Fig. 1a–c) under various growing conditions were weighed using an electronic scale (Uni-Bioc, China).

#### LC–MS sample preparation

Fully matured ripened fresh African horned cucumber fruit were harvested and promptly sent to the laboratory for analysis from each irrigation water level, soil type, and growing environments (Fig. 1d). The ripeness of the fruit was determined using the fruit colour chart developed by Nambi et al. (2015). The fruit samples were freeze-dried for 72 h using a freeze drier (HARVEST-RIGHT, Barcelona). Primary metabolites were extracted following the protocol of Kim and Verpoorte (2010). Briefly, 1.5 mL of MeOH (75% MEOH/25% water) was added on 0.5 g of freeze-dried ground fruit powder material and mixed using a vortex mixture. The mixture was sonicated for 5 min using the sonicate, (BRANDSON 1800 (Germany). The sonicated supernatant concentrate was then filtered through 0.2-micron syringe filters with 1 mL pipette. The supernatant filtered concentrate was then centrifuged in an Eppendorf tube (Centrifuge 5424, South Africa) at 10 000 revolutions per minute.

The supernatant concentrate of 72 samples was then transferred into the HLPC vials for LCMS-8040 triple quadrupole mass spectrometer, (Shimadzu, Japan) for analysis. The LCMS settings were as follows: total flow = 0.4 ml/min, injection volume = 1uL, oven temperature = 40 °C/max 85 °C, nebulising gas flow = 3L, drying gas flow = 15 L/min, and the mobile process was 50%/50% acetonitrile/water. To compare the treatments, the quantities of metabolites were plotted in Excel. The extracts were analysed by reverse phase LC–MS for their metabolomic contents. The MS analysis were carried out in electron spray (ESI) negative mode. It is worth to note that out of 99 primary metabolites, only a**s**paragine, dopa, 4-hydroxyproline, norepinephrine, niacinamide, kynurenine, 5-glutamylcysteine and acetylcarnitine were detected in all samples.

### Statistical analysis

Data gathered in this research was statistically analysed using three-way ANOVA, and the multiple-range Duncan test was used to separate treatment means, with statistical analysis software Statistica v. 10, StatSoft (USA. Values with p ≤ 0.05 were considered significant. The final statistical analysis was carried out using (Past 4) version 3 of 2013 and metaboanalyst 5.0.The software ((Past 4) version 3 of 2013) employs data normalization with functions such as data manipulation, univariate and multivariant statistics, whereas (metaboanalyst 5.0) is known as a comprehensive tool suitable for metabolic analysis that employs data normalization and also multivariant analysis methods such as (PCA and PLS-DA).

## Results

### Chlorophyll content and stomatal conductance

Table 1 shows how the chlorophyll content of African horned cucumber is affected by the combination of irrigation water stress and soil types in different growing environments. The interaction of irrigation water stress level and sandy loam soil under diverse growing environments produced substantial differences, according to the findings. The concentrations of chlorophyll ranged from 23.3 to 71.7 µmol m^−2^. In addition, the results showed that treatment interaction of sever water stress and loamy soil under shade net environment during season one reduced chlorophyll content from 71.7 to 20.6 µmol m^−2^ during preflowering stage, but no water stress level (control) and sandy loam soil in a open field environment raised it from 20.6 to 71.1 µmol m-^2^ during season the same season under flowering stage.

**Table 1:** Treatment interaction effect on the chlorophyll content (µmol m^−2^) of African horned cucumber.

2017/2018				2018/2019		
Treatment	Preflowering	Flowering	Fruiting	Preflowering	Flowering	Fruiting
**Chlorophyll content (µmol m**^**−2**^**)**						
W1S1L1	31.4 (0.1)	37.4 (1.3)	47.8 (1.1)	32.9 (1.1)	41.1 (1.1)	58.1 (1.0)
W1S1L2	32.9 (0.0)	41.3 (1.1)	55.8 (1.1)	37.9 (1.3)	45.5 (1.2)	66.2 (1.3)
W1S1L3	35.8 (0.1)	42.4 (0.1)	64.6 (1.0)	37.4 (0.3)	46.5 (1.1)	67.1 (1.1)
W1S2L1	29.2 (0.2)	26.4 (1.1)	46.7 (1.1)	27.6 (1.1)	49.4 (1.2)	60.2 (1.3)
W1S2L2	32.9 (0.1)	41.3 (0.1)	63.5 (0.0)	36.8 (2.3)	48.8 ( (1.1)	70.5 (1.2)
W1S2L3	36.8 (0.0)	45.8 (1.1)	71.7 (0.1)	39.6 (1.2)	43.9 (1.2)	73.8 (1.2)
W2S1L1	26.1 (1.1)	37.2 (0.1)	51.6 (0.0)	34.4 (0.2)	47.5 (1.2)	61.4 (1.2)
W2S1L2	27.1 (0.1)	36.1 (0.0)	37.2 (1.1)	34.7 (1.1)	46.2 (1.2)	52.4 (1.4)
W2S1L3	36.4 (1.1)	44.7 (1.1)	46.3 (0.1)	39.6 (1.3)	49.5 (1.2)	64.7 (1.1)
W2S2L1	31.9 (1.0)	41.4 (0.1)	50.3 (1.1)	34.4 (1.5)	49.4 (1.1)	65.9 (1.2)
W2S2L2	25.6 (1.1)	37.9 (0.1)	43.1 (0.0)	34.7 (1.1)	50.1 (1.1)	61.5 (1.2)
W2S2L3	34.1 (0.0)	36.8 (1.1)	46.4 (1.1)	39.3 (1.2)	53.1 (1.1)	58.8 (1.1)
W3S1L1	22.9 (1.1)	25.7 (0.1)	40.7 (1.1)	27.9 (1.4)	48.9 (1.1)	44.4 (1.2)
W3S1L2	20.6 (0.1)	24.9 (0.0)	35.3 (0.1)	29.5 (1.2)	40.2 (1.0)	46.5 (1.1)
W3S1L3	25.7 (1.1)	25.2 (1.1)	40.6 (1.2)	39.3 (1.4)	46.3 (1.2)	44.8 (1.1)
W3S2L1	23.3 (0.1)	26.4 (0.1)	36.6 (1.2)	27.6 (1.4)	47.6 (1.1)	45.8 (1.2)
W3S2L2	23.3 (1.1)	24.9 (1.1)	36.4 (1.0)	29.5 (1.2)	42.5 (1.2)	42.2 (1.2)
W3S2L3	23.8 (0.0)	24.8 (1.1)	36.6 (1.3)	33.3 (1.1)	54.2 (1.4)	43.87 (1.1)
Grand mean	**41.44**	**41.44**	**41.44**	**41.44**	**41.44**	**41.44**
LSD_0.05_	**6.706**	**6.706**	**6.706**	**6.706**	**6.706**	**6.706**
P_value_	**0.042**	**0.042**	**0.042**	**0.042**	**0.042**	**0.042**

Treatment interaction effect on the chlorophyll content of African horned cucumber.

*W1* no water stress, *W2* moderate water stress, *W3* severe water stress, *S1* loamy soil, *S2* sandy loam, *L1* greenhouse, *L2* shade net, *L3* open field.

Numbers in brackets represent the standard deviations of the mean. LSD_0.05_ is the least significant difference of means. Years (seasons one—2017/18 and season two—2018/2019).

P values in bold are lower than 0.05.

The results in (Table 2) on the African horned cucumber stomatal conductance demonstrated a substantial interaction between different water stress levels and soil types under varying growing environment during different seasons. Stomatal conductance was measured and found to be between 34.8 and 67.6 mmol m^−2^. In a greenhouse environment, severe water stress and sandy loam soil resulted in a decrease from 67.6 to 34.8 mmol m^−2^, during season one [2017/2018], but moderate water stress and sandy loam soil during the same season [2017/18], resulted in an increase from 34.8 to 67.6 mmol m^−2^ in an open field environment.

**Table 2:** Treatment interaction effect on the stomatal conductance (mmol m^-2^) of African horned cucumber.

2017/2018				2018/2019		
Treatment	Preflowering stage	Flowering stage	Fruiting stage	Preflowering stage	Flowering stage	Fruiting stage
**Stomatal conductance (mmol m**^**−2**^**)**						
W1S1L1	41.2 (1.1)	47.1 (1.1)	40.2 (1.1)	43.2 (1.4)	46.5 (1.2)	44.2 (1.1)
W1S1L2	46.4 (1.2)	49.3 (0.0)	37.1 (2.3)	42.2 (0.2)	48.2 (1.1)	35.1 (1.4)
W1S1L3	41.2 (0.5)	47.2 (1.3)	36.9 (1.4)	40.4 (1.1)	45.7 (0.4)	37.2 (2.2)
W1S2L1	39.0 (1.1)	50.0 (1.2)	38.8 (0.5)	36.2 (1.3)	47.3 (1.4)	36.3 (1.4)
W1S2L2	45.3 (1.1)	56.2 (1.2)	38.1 (1.1)	42.5 (1.4)	51.0 (1.1)	34.9 (0.4)
W1S2L3	43.7 (1.3)	46.0 (1.4)	36.4 (1.3)	40.4 (2.3)	46.2 (1.3)	35.1 (2.1)
W2S1L1	40.7 (1.2)	51.5 (1.3)	36.7 (1.1)	44.3 (1.1)	50.4 (0.7)	36.2 (3.2)
W2S1L2	41.9 (1.1)	45.6 (1.3)	38.3 (1.4)	43.6 (1.4)	48.2 (1.2)	37.3 (1.1)
W2S2L3	40.7 (1.3)	44.9 (1.6)	38.4 (1.7)	41.8 (1.3)	42.6 (1.1)	34.3 (1.4)
W2S2L1	43.3 (1.2)	50.3 (1.1)	37.4 (1.2)	39.4 (1.4)	52.4 (1.3)	37.4 (1.2)
W2S2L2	41.6 (1.1)	44.2 (1.3)	39.3 (1.6)	44.3 (1.3)	45.3 (1.1)	36.6 (3.2)
W2S2L3	41.6 (1.4)	43.0 (1.1)	67.6 (1.4)	38.4 (1.1)	44.3 (2.4)	59.7 (2.1)
W3S1L1	37.1 (1.5)	38.7 (1.4)	37.4 (1.0)	35.5 (1.2)	39.4 (1.6)	35.7 (1.4)
W3S1L2	36.4 (1.3)	35.9 (1.1)	38.0 (0.0)	37.6 (2.3)	33.5 (2.3)	36.7 (2.2)
W3S1L3	36.5 (1.3)	41.8 (1.3)	35.5 (1.4)	34.3 (0.8)	39.7 (2.4)	37.4 (2.1)
W3S2L1	34.8 (1.2)	37.6 (1.4)	37 (0.1)	39.3 (1.2)	34.2 (1.4)	36.4 (1.1)
W3S2L2	36.9 (1.3)	37.9 (1.1)	36.0 (1.2)	34.2 (1.1)	34.2 (1.5)	36.5 (2.2)
W3S2L3	38.2 (1.4)	35.2 (1.3)	36.7	38.2 (0.5)	37.6 (2.2)	38.2 (1.4)
Grand mean	**41.41**	**41.41**	**41.41**	**41.41**	**41.41**	**41.41**
LSD_0.05_	**4.329**	**4.329**	**4.329**	**4.329**	**4.329**	**4.329**
P_value_	**0.038**	**0.038**	**0.038**	**0.038**	**0.038**	**0.038**

Treatment interaction effect on the stomatal conductance of African horned cucumber.

*W1* no water stress, *W2* moderate water stress, *W3* severe water stress, *S1* loamy soil and S2 means sandy loam, *L1* greenhouse, *L2* shade net, *L3* open field, *WUE* water use efficiency.

Numbers in brackets represent the standard deviations of the mean. LSD_0.05_ is the least significant difference of means. Years (seasons one—2017/18 and season two—2018/2019).

P values in bold are lower than 0.05.

### Fresh fruit weight

Table 3 shows the impact of irrigation water stress, soil types, and growth environment on the fresh weight of African horned cucumber fruits over various seasons. According to the findings, there is no substantial difference between various levels of water stress, soil types, and growing conditions. Fresh fruit weights ranged from 104.1 to 686.1 g during season one, whereas 143.1 to 722.2 g during season two. During season two [2018/19], the treatment combination of no water stress and sandy loam soil produced the largest fruit weight (722.2 g), which was seen in a greenhouse environment. During season one [2017/18], the treatment with severe water stress and sandy loam soil in an open field environment yielded the smallest fresh fruit weight (104.1 g).

**Table 3:** Treatment interaction effect on the fresh fruit weight **(g)** of African horned cucumber.

Treatment	2017/18	2018/19
**Fresh fruit weight (g)**		
W1S1L1	686.1 (58)	679.5 (22)
W1S1L2	548.6 (159)	380.8 (41)
W1S1L3	221.7 (55)	305.7 (117)
W1S2L1	679 (49)	722.2 (122)
W1S2L2	449.1 (38)	357.2 (36)
W1S2L3	236 (29)	305.7 (43)
W2S1L1	540.2 (99)	345.3 (40)
W2S1L2	548.6 (164)	389.5 (25)
W2S1L3	305.1 (12)	325.2 (51)
W2S2L1	676.5 (8)	275.9 (26)
W2S2L2	197.9 (18)	389.5 (17)
W2S2L3	234.2 (18)	315 (28)
W3S1L1	397.4 (11)	284 (13)
W3S1L2	139.1 (14))	187.6 (23)
W3S1L3	106.1 (37)	143.1 (31)
W3S2L1	313.7 (5)	296.2 (22)
W3S2L2	139.1 (21)	218.1 (9)
W3S2L3	104.1 (8)	216.6 (12)
Grand mean	**342.1**	**342.1**
LSD_0.05_	**100.64**	**100.64**
P_value_	**0.096**	**0.096**

Treatment interaction effect on the fruit weight of African horned cucumber.

*W1* no water stress, *W2* moderate water stress, *W3* severe water stress, *S1* loamy soil and *S2* sandy loam, *L1* means greenhouse, *L2* means shade net, *L3* mean open field, *WUE* water use efficiency.

Numbers in brackets represent the standard deviations of the mean. LSD_0.05_ is the least significant difference of means. Years (seasons one—2017/18 and season two—2018/2019).

P values in bold are lower than 0.05.

### Primary metabolites

#### Asparagine

**Figure 2:**
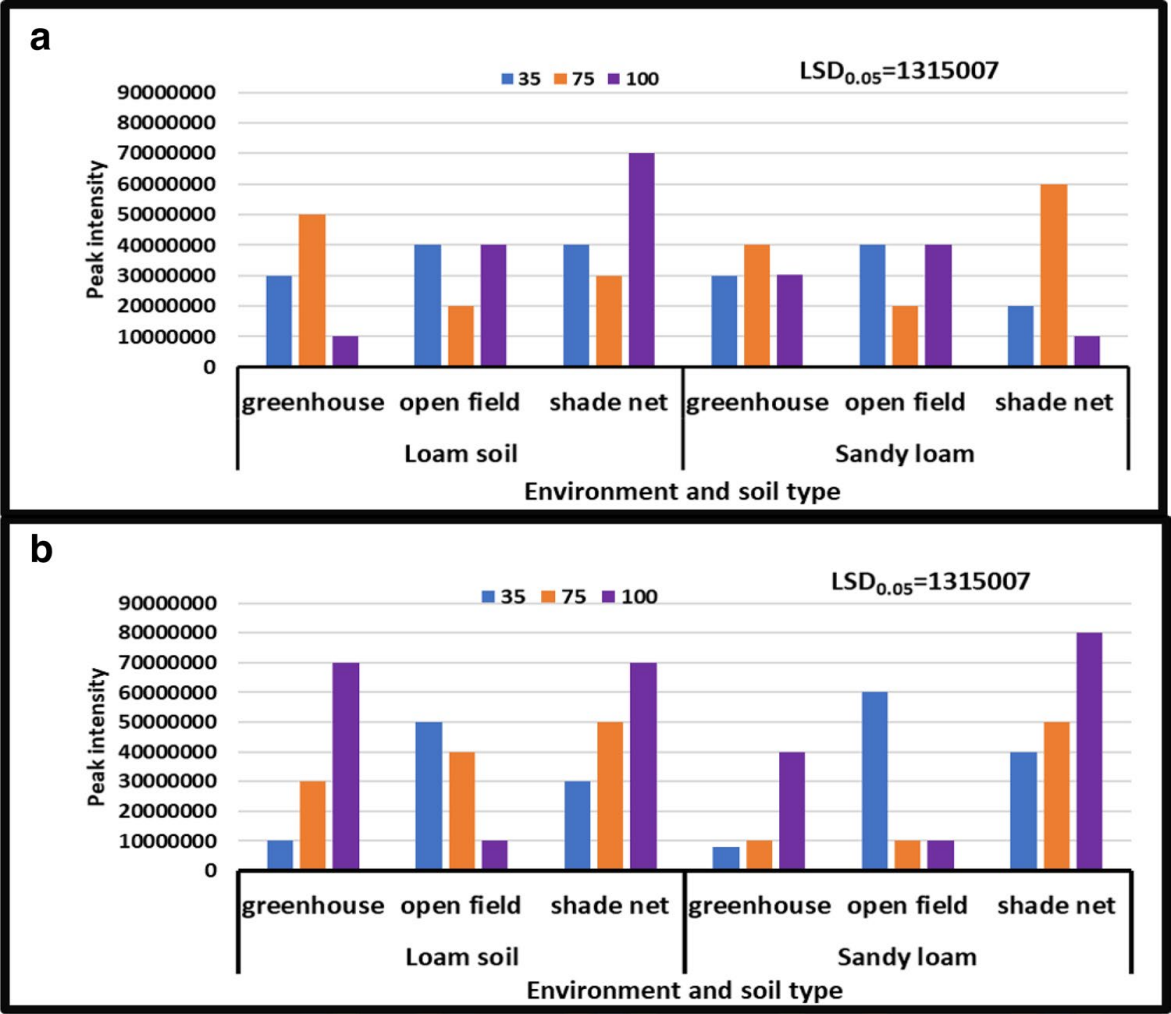
Treatment interaction effect on asparagine of African horned cucumber fruit. (**a**) Means treatment interaction of irrigation water regimes and soil types at different growing environments during the 2017/18 season; (**b**) means treatment interaction of water stress levels and soil types at different growing environments during the 2018/19 season. Values are average over treatment; 35 means severe water stress; 75 means moderate water stress; 100 means no water stress. Peak intensity showing LCMS-8040 triple quadrupole mass spectrometer intensities which represent the quantity of metabolites varying with treatments. LSD_0.05_ is the least significant difference of means.

According to the findings, the treatment interaction between different levels of water stress and distinct soil types had a significant impact on asparagine levels in different growing environments. In all growing environments, during the year [2018/19], the treatment with no water stress (control) showed higher asparagine content than the other treatments. During the 2017/18 season, asparagine content peaked at 10 × 10^6^ to 60 × 10^6^, while during the 2018/19 season, it peaked at 80 × 10^6^ to 10 × 10^6^. During the second season [2018/19], severe water stress in combination with sandy loam in a greenhouse environment significantly reduced asparagine from 80 × 10^6^ to 10 × 10^6^ peak intensity, whereas no water stress in combination with sandy loam in a shade net environment significantly increased it from 10 × 10^6^ to to 80 × 10^6^ peak intensity (Fig. 2).

#### Dopa

**Figure 3:**
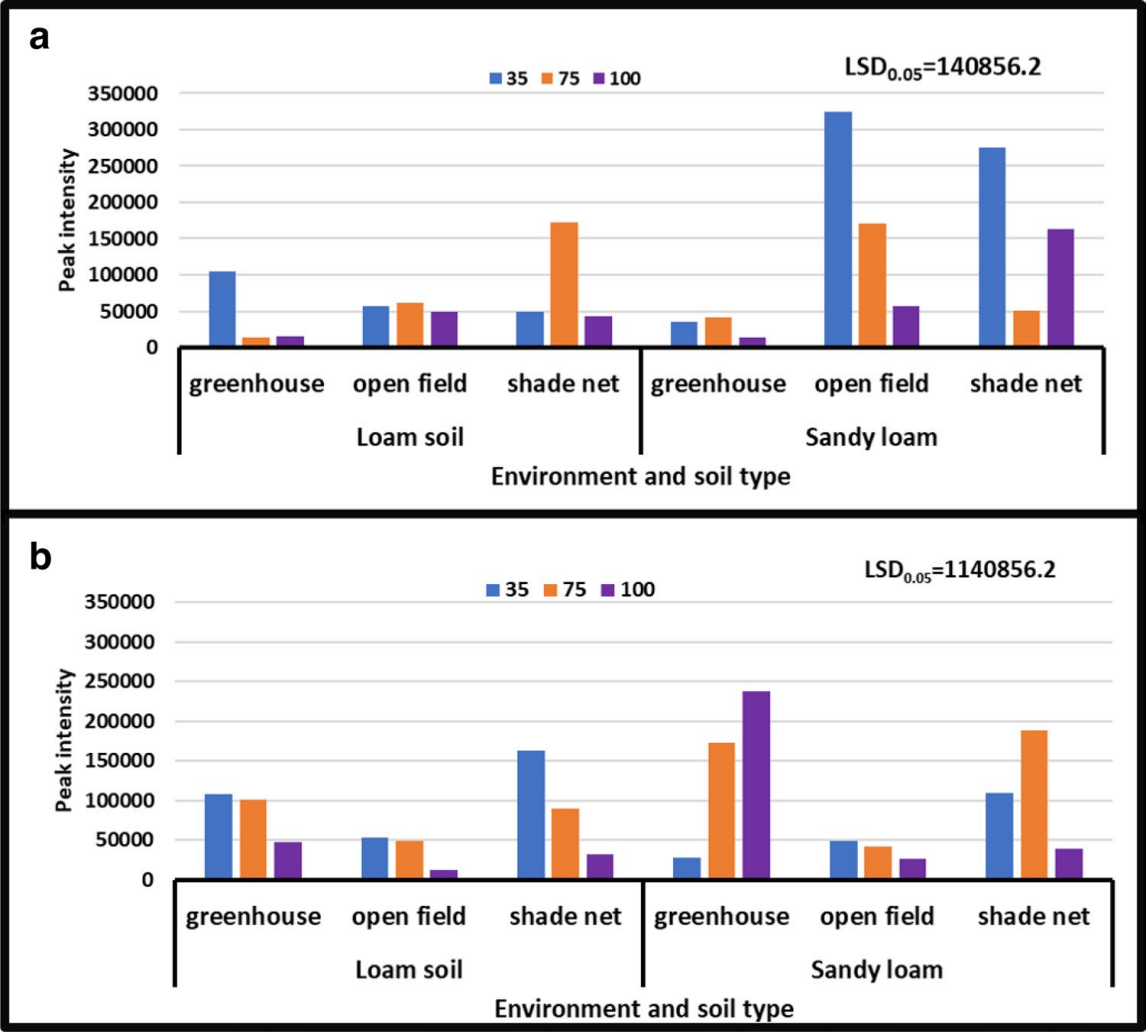
Treatment interaction effect on Dopa of African horned cucumber fruit. Values are average over treatment; 35 means severe water stress; 75 means moderate water stress; 100 means no water stress; (**a**) means treatment interaction of water stress levels and soil types at different environments during the 2017/18 season; (**b**) means treatment interaction of irrigation water regimes and soil types at different environments during the 2018/19 season. Peak intensity showing LCMS-8040 triple quadrupole mass spectrometer intensities which represent the quantity of metabolites varying with treatments. LSD_0.05_ is the least significant difference of means.

During both seasons, there was a substantial difference in the interaction between irrigation water levels and soil types, according to the study's findings. Dopa content ranged from 13,697 to 324, 240 peak intensity in the year [2017/18], while it ranged from 12,030 to 47,611 peak intensity in the year [2018/19]. During the year [2018/19], the no water stress treatment (control) in combination with loamy soil in open field environment reduced dopa content from 324,240 to 12,030 peak intensity. During the year [2017/18], treatment of severe water stress in combination with sandy loam soil in open field environment increased peak intensity from 12,030 to 324,240 (Fig. 3).

#### 4-Hydroxyproline

**Figure 4:**
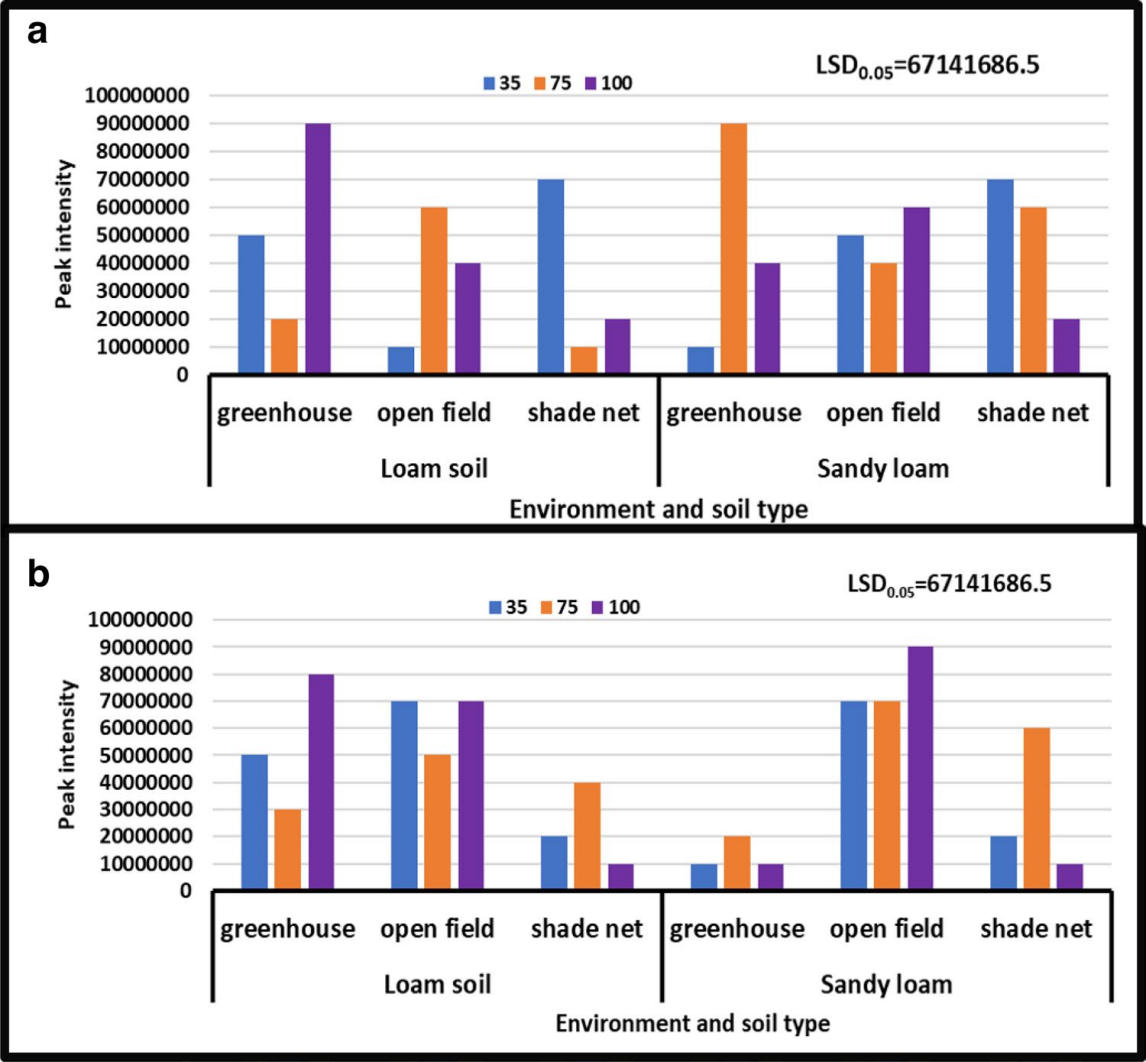
Treatment interaction effect on 4-hydroxyproline of African horned cucumber. 35 means severe water stress; 75 means moderate water stress; 100 means no water stress. Values are average over treatment; (**a**) means treatment interaction of water stress levels and soil types at different environments during the 2017/18 season; (**b**) means treatment interaction of irrigation water regimes and soil types at different environments during season 2018/19. Peak intensity showing LCMS-8040 triple quadrupole mass spectrometer intensities which represent the quantity of metabolites varying with treatments. LSD_0.05_ is the least significant difference of means.

During both seasons, the results revealed no significant differences in the interaction of irrigation water levels and soil types at different growing environments. However, the content of 4-hydroxyproline varied significantly depending on the growth environment. During the year [2017/18], peak intensity of 4-hydroxyproline varied from 10 × 10^6^ to 90 × 10^6^, whereas peak intensity of 4-hydroxyproline ranged from 10 × 10^6^ to 90 × 10^6^ during the year [2018/19]. Treatment of all irrigation water levels combined with both soil types under all growing conditions during the years [2017/18] and [2018/19] under shade net and greenhouse (Fig. 4b) increased 4-hydroxyproline content from 90 × 10^6^ to 10 × 10^6^ peak intensity, whereas treatment of irrigation water levels combined with both soil types under greenhouse environment during the year [2017/18] and treatment of no water stress combined with sandy loam soil during the year [2018/19] decreased 4-hydroxyproline content from 10 × 10^6^ to 90 × 10^6^ peak intensity, respectively (Fig. 4a,b).

#### 5-Glutamylcysteine

**Figure 5:**
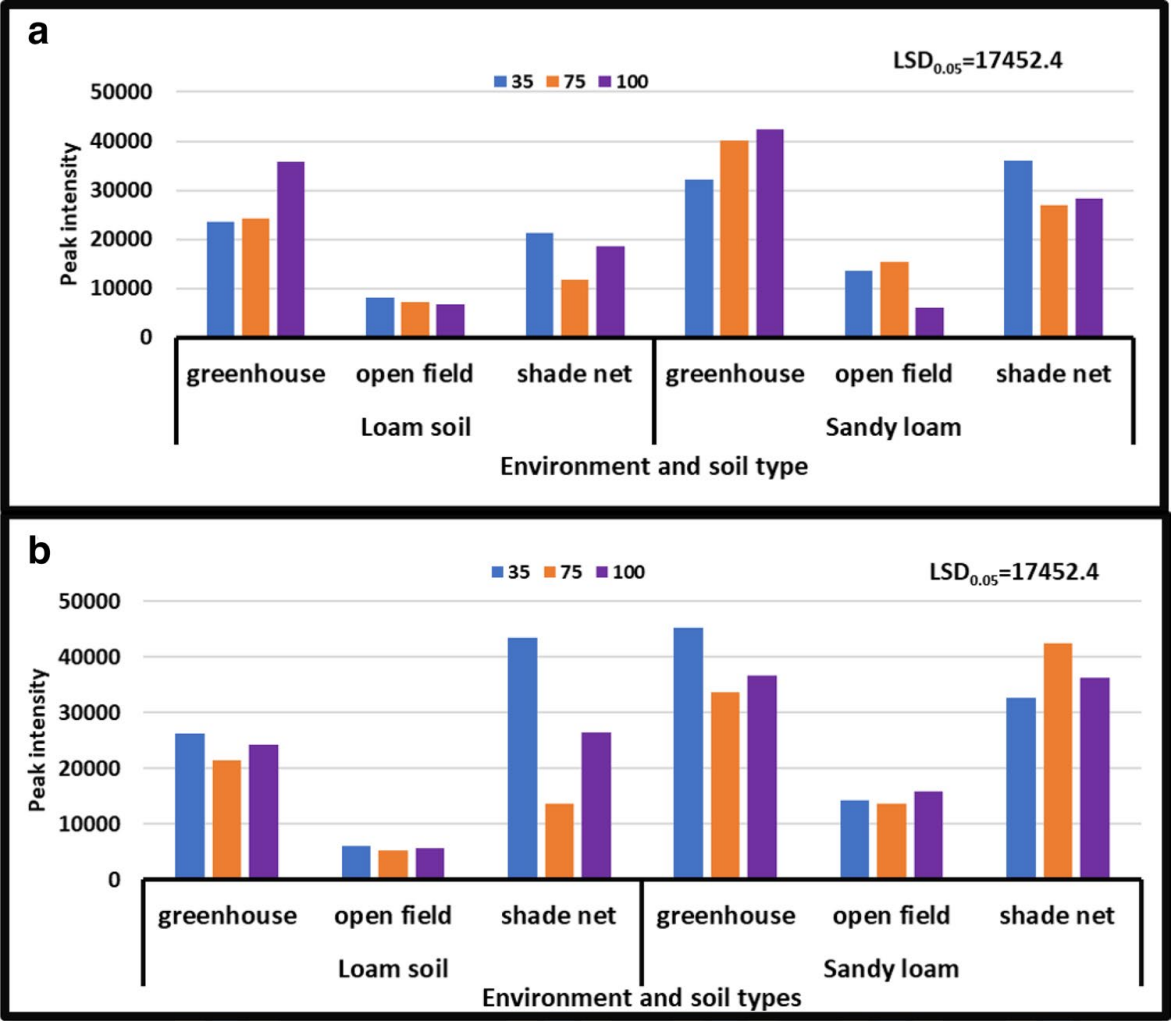
Treatment interaction effect on 5-glutamylcusteine of African horned cucumber fruit. 35 means severe water stress; 75 means moderate water stress; 100 means no water stress; (**a**) means treatment interaction of water stress levels and soil types at different growing environments during the 2017/18 season; (**b**) means treatment interaction of irrigation water regimes and soil types at different growing environments during the 2018/19 season. Values are average over treatment. Peak intensity showing LCMS-8040 triple quadrupole mass spectrometer intensities which represent the quantity of metabolites varying with treatments. LSD_0.05_ is the least significant difference of means.

The findings demonstrated that there was a substantial difference in 5-glutamylcysteine concentration when irrigation water levels and soil types were combined in different growing environments during all seasons. 5-glutamylcysteine content ranged from 6115 to 35,818 peak intensity in the year [2017/18], while it ranged from 5345 to 45,214 peak intensity in the year [2018/19]. During the year [2018/19], the treatment of moderate water stress combined with loam soil in an open field environment resulted in a considerable decrease in 5-glutamylcysteine concentration, which decreased from 45,214 to 6115 peak intensity (Fig. 5b). During the year [2018/19], the extreme water stress treatment combined with sandy loam under greenhouse environment resulted in a considerable rise in 5-glutamylcysteine content, which increased from 6, 115 to 45,214 peak intensity (Fig. 5).

#### Acetylcarnitine

**Figure 6:**
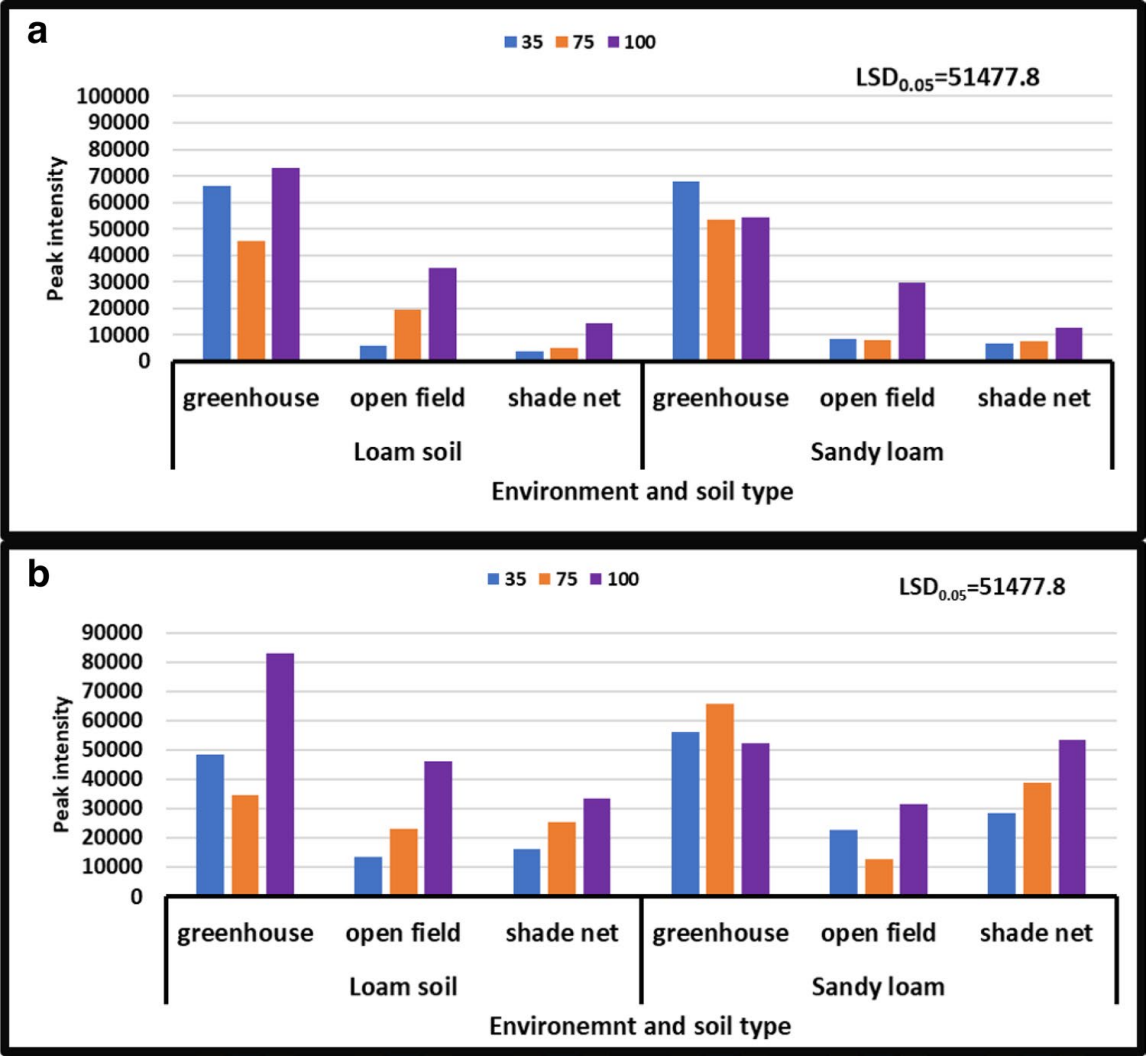
Treatment interaction effect on Acetylcarnitine of African horned cucumber. 35 means severe water stress; 75 means moderate water stress; 100 means no water stress; (**a**) means treatment interaction of water stress levels and soil types at different growing environments during the 2017/18 season one; (**b**) means treatment interaction of irrigation water levels and soil types at different growing environments during the 2018/19 season. Values are average over treatment. Peak intensity showing LCMS-8040 triple quadrupole mass spectrometer intensities which represent the quantity of metabolites varying with treatments. LSD_0.05_ is the least significant difference of means.

During both seasons, the results revealed a considerable variance in irrigation water levels and soil types at different growing environments. The observed trend revealed that acetylcarnitine concentration was higher in the year [2017/18] than in the year [2018/19]. Acetylcarnitine content ranged from 3761 to 72, 841 peak intensity in 2017/18, whereas it ranged from 12,514 to 82,841 peak intensity in 2018/19. The results in Fig. 6a, also showed that treatment of severe water stress with loamy soil in a shade net environment significantly reduced acetylcarnitine content from 82,841 to 3761 peak intensity during the [2017/18], whereas results in Fig. 6b, delineated that treatment of no water stress (control) with loamy soil in a greenhouse environment significantly increased it from 3761 to 82,841 peak intensity during the [2018/19].

#### Kynurenine

**Figure 7:**
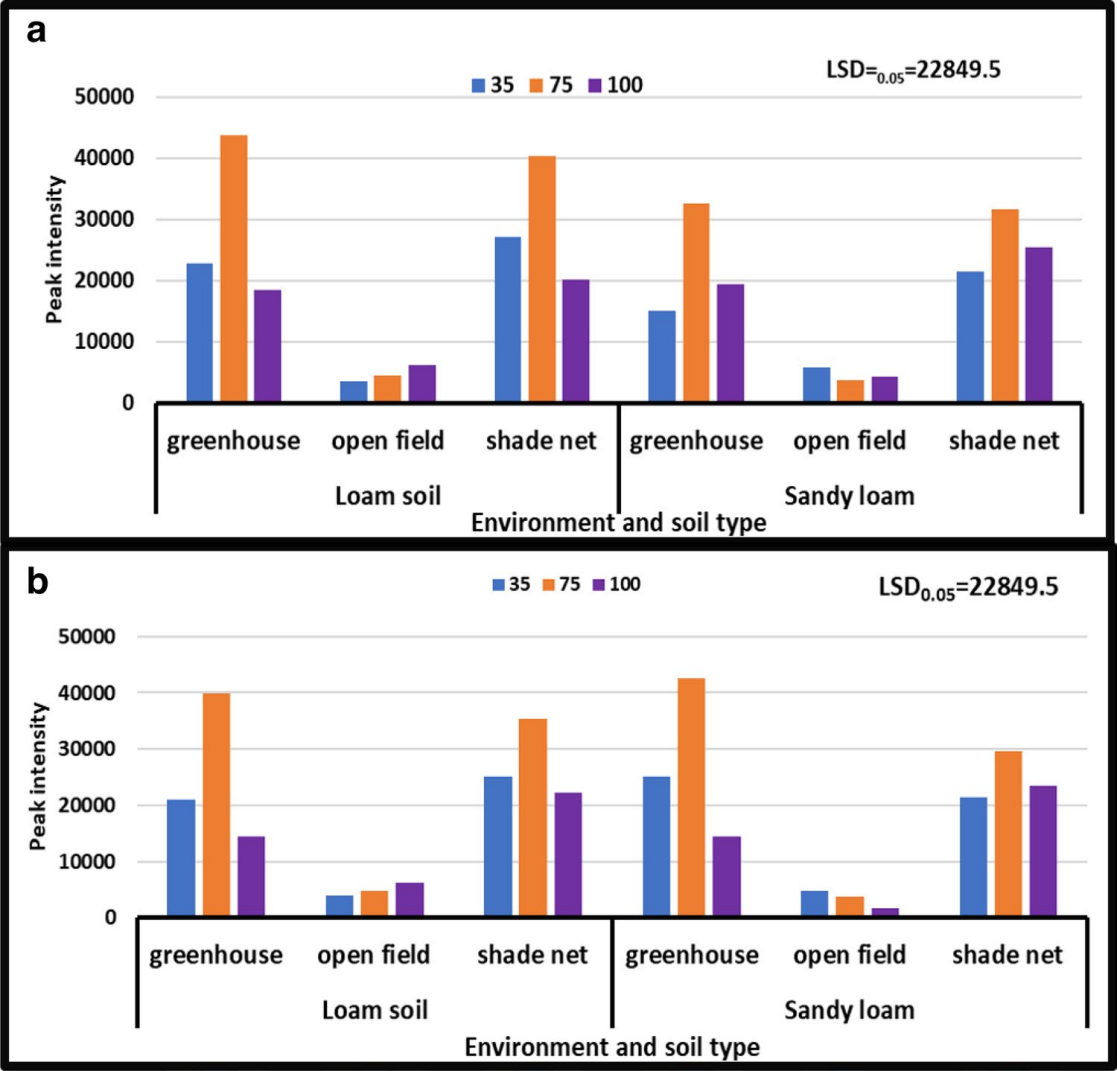
Treatment interaction effect on Kynurenine of African horned cucumber fruit. 35 means severe water stress; 75 means moderate water stress; 100 means no water stress; (**a**) means treatment interaction of irrigation water levels and soil types at different growing environments during the 2017/18 season; (**b**) means treatment interaction of irrigation water levels and soil types at different growing environments during the 2018/19 season. Values are average over treatment. Peak intensity showing LCMS-8040 triple quadrupole mass spectrometer intensities which represent the quantity of metabolites varying with treatments. LSD_0.05_ is the least significant difference of means.

During both seasons, the study found a substantial variation in kynurenine concentration when irrigation water levels and soil types were combined in distinct growing environments (Fig. 7). The severe stress treatment had a larger kynurenine concentration than the other irrigation water levels, according to the observed pattern. It ranged from 3602 to 43,808 peak intensity in the year [2017/18], while it ranged from 1678 to 42,555 peak intensity in the year [2018/19]. During the year [2018/19], the no water stress treatment (control) combined with sandy loam soil in an open field environment showed a significant decrease in kynurenine content from 43,808 to 1678 peak intensity (Fig. 7b), whereas the treatment of moderate water stress combined with loamy soil in a greenhouse environment showed an increase from 1,678 to 43,808 peak intensity (Fig. 7a).

#### Norepinephrine

**Figure 8:**
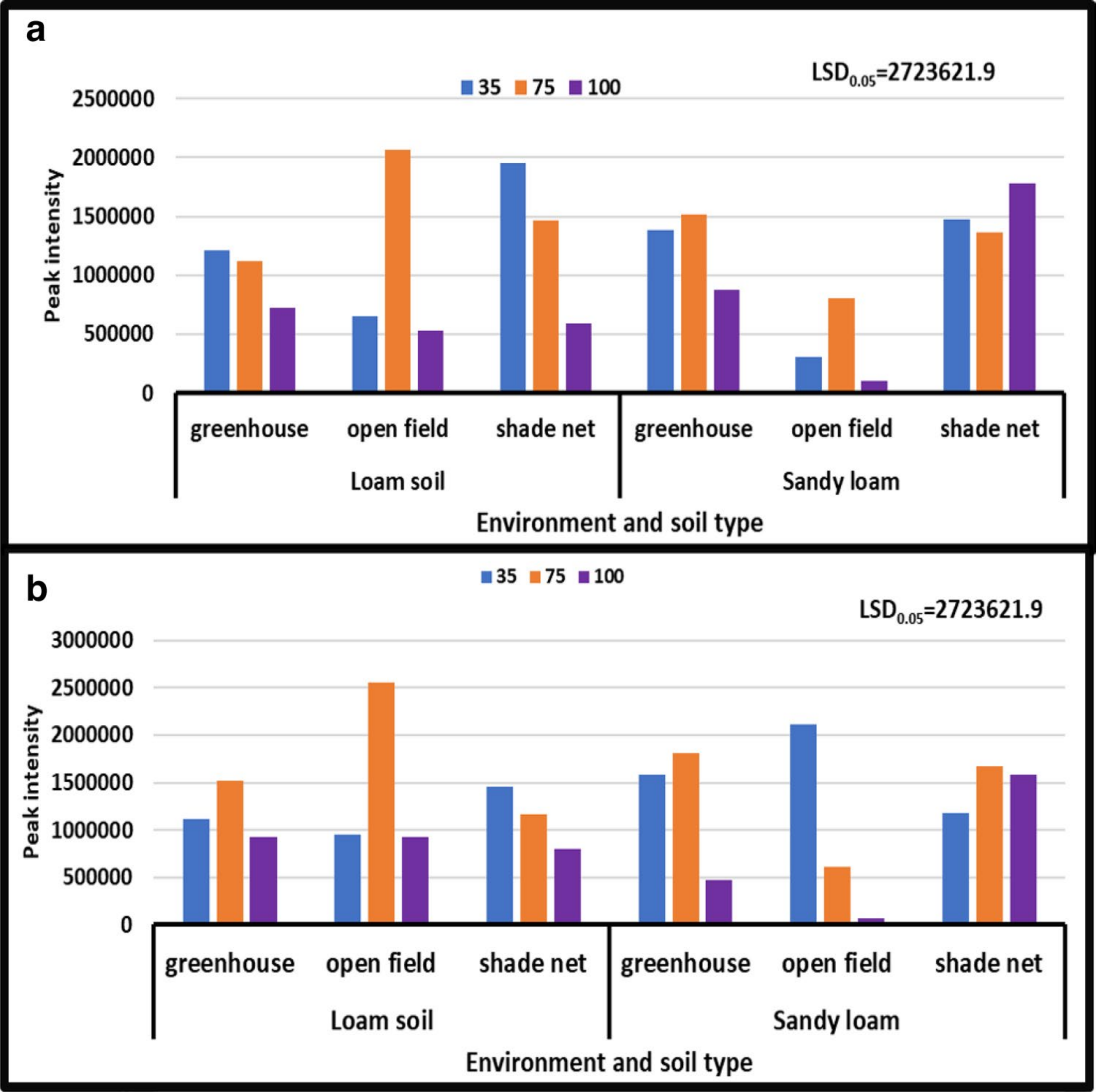
Treatment interaction effect on Norepinephrine of African horned cucumber fruit. 35 means severe water stress; 75 means moderate water stress; 100 means no water stress; (**a**) means treatment interaction of water stress levels and soil types at different growing environments during the 2017/18 season; (**b**) means treatment interaction of irrigation water levels and soil types at different growing environments during the 2018/19 season. Values are average over treatment. Peak intensity showing LCMS-8040 triple quadrupole mass spectrometer intensities which represent the quantity of metabolites varying with treatments. LSD_0.05_ is the least significant difference of means.

The results showed that there was no significant difference in norepinephrine content at different growing environments when irrigation water levels and soil types were combined. However, there was a considerable variation in the results under different growing environment. Norepinephrine levels varied from 99,577 to 206, 1045 peak intensity in the 2017/18 year, and from 71,577 to 256, 1045 peak intensity in the 2018/19 year. The no water stress treatment (control) in combination with sandy loam soil in an open field environment reduced norepinephrine content from 256,1045 to 71,577 peak intensity (Fig. 8b), whereas the moderate water stress treatment in combination with loamy soil significantly increased it from 71,577 to 256,1045 peak intensity (Fig. 8).

#### Niacinamide

**Figure 9:**
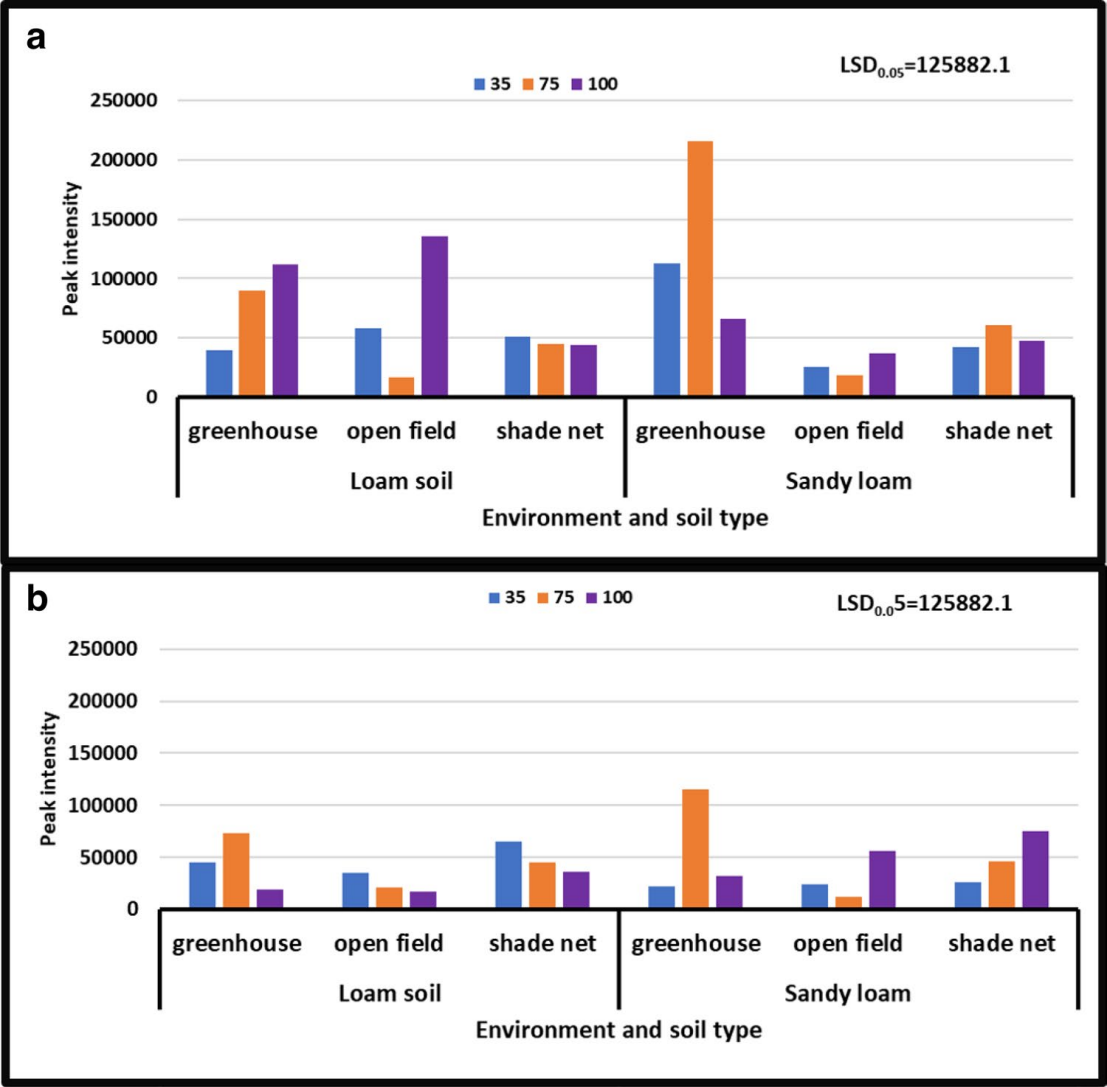
Treatment interaction effect on Niacinamide of African horned cucumber fruit. 35 means severe water stress; 75 means moderate water stress; 100 means no water stress; (**a**) means treatment interaction of irrigation water regimes and soil types at different growing environments during the 2017/18 season; (**b**) means treatment interaction of water stress levels and soil types at different growing environments during the 2018/19 season. Values are average over treatment. Peak intensity showing LCMS-8040 triple quadrupole mass spectrometer intensities which represent the quantity of metabolites varying with treatments. LSD_0.05_ is the least significant difference of means.

During both seasons, the results revealed a considerable difference in the interaction of irrigation water levels and soil types under distinct growing environments. Niacinamide content ranged from 16,318 to 216,137 peak intensity in the 2017/18 year, and from 12,521 to 115,147 peak intensity in the 2018/19 year. Results in (Fig. 9b), showed that the treatment of moderate water stress combined with sandy loam in an open field environment decreased niacinamide content from 216,137 to 12,521 peak intensity during the year [2018/19]. During the year [2017/18], the moderate water stress treatment combined with sandy loam in a greenhouse environment dramatically enhanced the peak intensity of this compound from 12,521 to 216,137 (Fig. 9).

### LC–MS primary metabolomic analysis using PCA analysis

**Figure 10:**
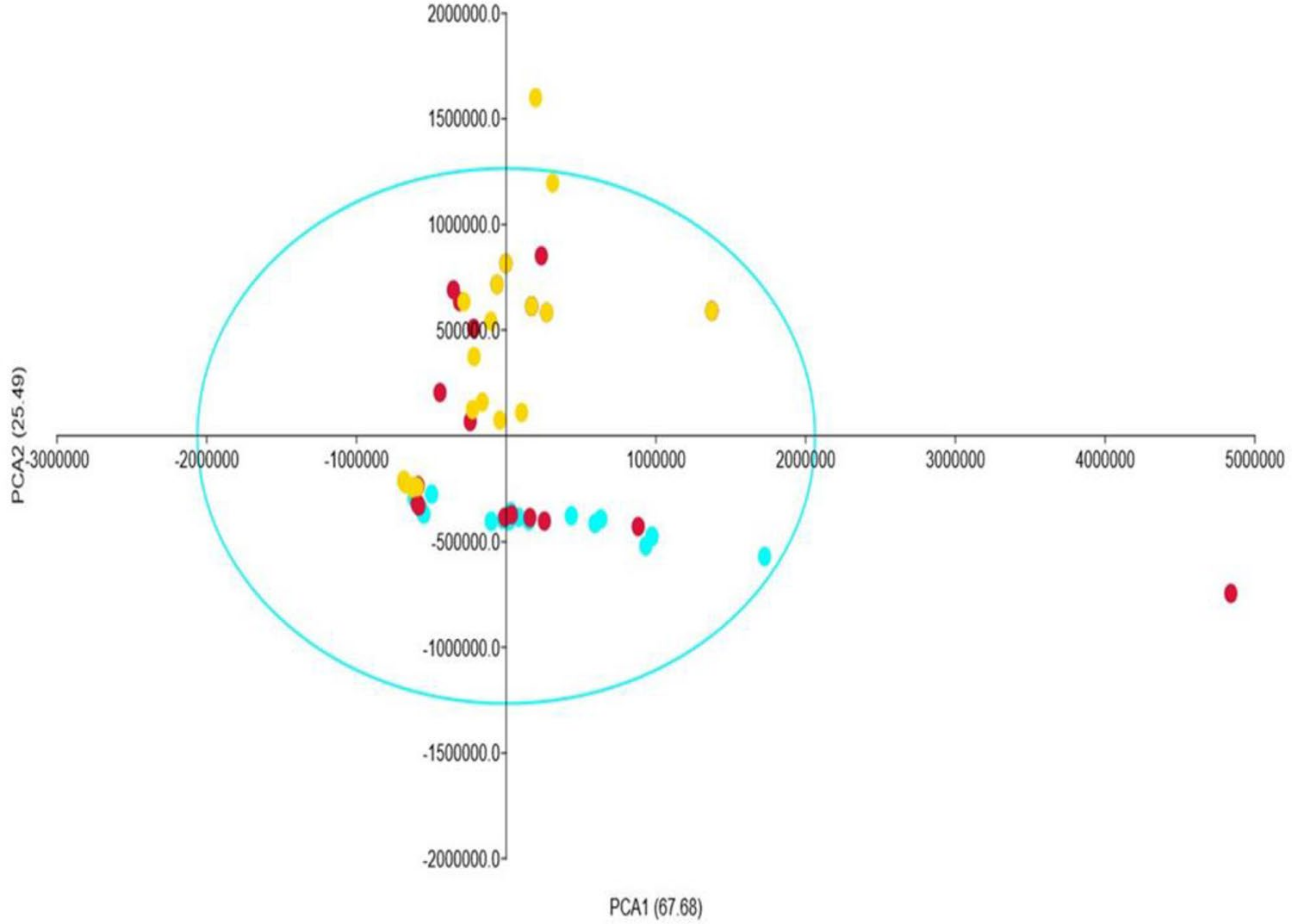
Principal component analysis of primary metabolites profile of African horned cucumber fruit harvested from different treatments. The explained variables are shown in different colours (Blue = Greenhouse, Red = shade net, and Yellow = open field).

All fruit samples' data was evaluated, and a distinct separation was achieved (Fig. 10). To achieve proper interpretation of primary metabolites data, autoscaling data pre-treatment was applied. The samples taken in the open field environment showed the most variances from those collected in the greenhouse and shade net environments. When contributing plots were built, it became obvious that the quantity of metabolites in greenhouse and shade net fruit was not considerably separated, as compared to those of open field environment. In comparison to fruit gathered in the open field environment, the metabolites in the greenhouse and shade net did not exhibit a clear distinction. However, when metabolites from open fields were compared to those from greenhouses and shade nets, the contribution plot clearly shows that they were clearly separated (Fig. 10).

### Important features of African horned cucumber fruit metabolites (VIP scores identified by PLS-DA)

**Figure 11:**
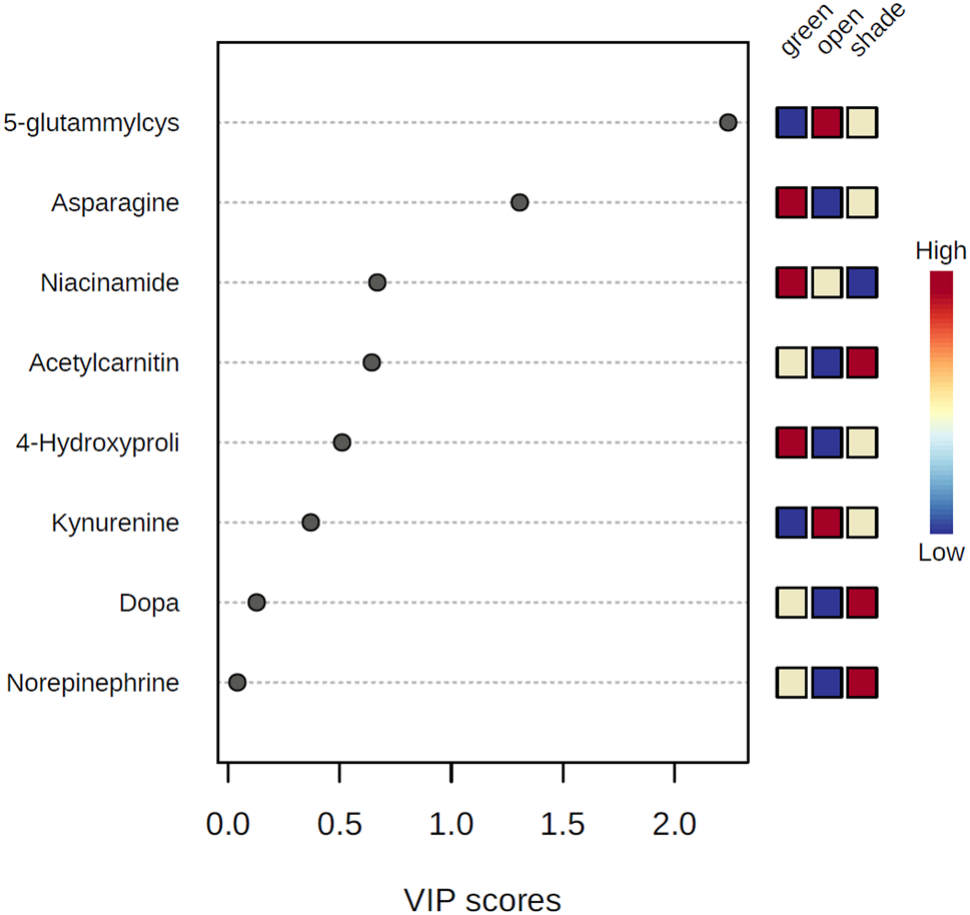
VIP scores of year one [2017/18] primary metabolites of African horned cucumber identified by PLS-DA. The coloured boxes on the right indicate the relative concentrations of the corresponding metabolite in each growing environment (greenhouse, shade net and open field).

**Figure 12:**
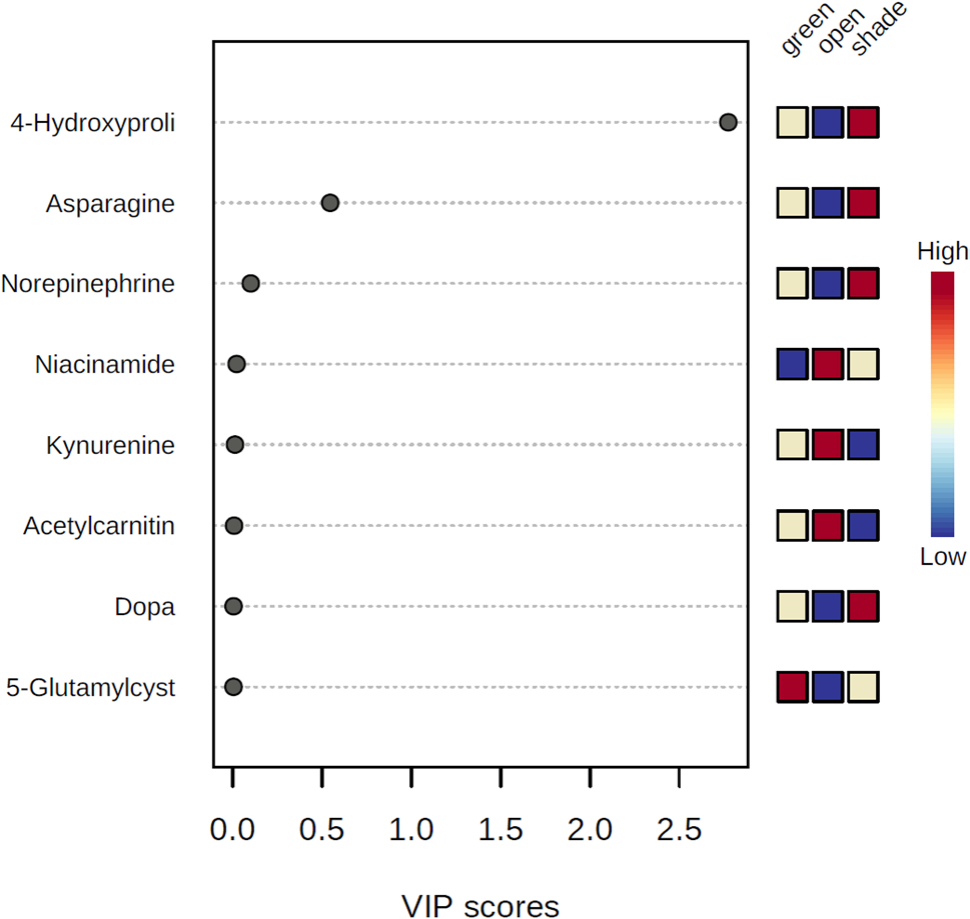
VIP scores of year two [2018/19] primary metabolites of African horned cucumber identified by PLS-DA. The coloured boxes on the right indicate the relative concentrations of the corresponding metabolite in each growing environment (greenhouse, shade net and open field).

All African horned cucumber metabolite had a cumulative VIP value of more than 1.5. During the year (2017/18), studied samples revealed that 5-glutamylsteine and kynurenine content were higher in open space environment, whereas asparagine, niacinamide, and 4-hydroxyproline content were superior in greenhouse environment. Moreover, the abundance of acetylcarnitine, dopa, and norepinephrine was higher under the shade net (Fig. 11). In addition, the study results showed that the concentration of 4-hydroxyproline, asparagine, norepinephrine, and dopa was higher in the shade net environment compared to other growing environments, whereas niacinamide, kynurenine, and acetylcarnitine was significantly concentrated in the open field environment during the year (2018/19). The abundance of 5-gluatamylcystein was higher in the greenhouse when compared to other growing environments (Fig. 12).

## Discussion

The metabolites profile changes of African horned cucumber were investigated under various water stress levels and soil types in diverse growing environments. To our knowledge, this is the first study to look at metabolite alterations in African horned cucumber, therefore the results serve as a baseline. The findings of this study revealed that in a greenhouse environment, the interaction of water stress levels (no water stress, moderate water stress, and moderate water stress) and soil types (loamy soil and sandy loam soil) resulted in a significant increase in the content of most primary metabolites in African horned cucumber fruit, whereas some primary metabolites were significantly affected by either water stress levels or soil types in different environments.

The results of the study revealed that sandy loam soil had a higher chlorophyll content than loamy soil. However, there was unequivocal evidence that severe water stress resulted in a significant reduction in chlorophyll concentration when compared to moderate and no water stress levels. Plants grown in open spaces had greater chlorophyll mean values (71.7 µmol m^−2^), whereas those planted in greenhouses had the lowest (23.3 µmol m^−2^). The difference between the greatest and lowest chlorophyll concentration values was (48.4 µmol m^−2^). Water stress and location were found to be contributors to diversity in chlorophyll concentration among plants, according to the findings. These differences could be due to osmoregulation imbalances generated by differing amounts of water stress and environmental circumstances, which then affect the xylem and phloem functions, which are critical in controlling photosynthesis and crop performance. Several investigations on the effect of water levels on the photosynthetic process have revealed that the chlorophyll concentration of plants reduces in response to water stress. Low moisture in plants reduces net photosynthesis by causing stomatal closure, which leads to metabolic defects, according to Tona et al.^26^.

Open field environments had higher (67.6 mmol m^−2^) stomatal conductance than shade net environments (33.5 mmol m^−2^), according to the findings. The fact that there was (34.3 mmol m^−2^) stomatal difference between treatments could be a strong evidence to prove that treatment imposed play a crucial role in plant metabolic activities. When comparing treatments, moderate water stress combined with sandy loam resulted in higher stomatal conductance, but severe water stress resulted in lower stomatal conductance. This shows that as plant root water intake decreases, plant physiological performance declines, resulting in stomatal closure and possibly affecting metabolite levels and functions. We can expect a reduction in osmoregulation when plants are exposed to water stress conditions because the phloem is unable to provide sufficient solutes needed by the crop to meet transpiration demand, as reported by Legwaila et al.^27^, who found lower stomatal conductance on crops exposed to lower irrigation frequencies compared to crops exposed to higher irrigation frequencies.

There was no significant interaction between treatments in terms of fresh fruit weight, according to the study data. Despite the fact that the interaction was not statistically significant, the means revealed that in an open field environment, fresh fruit weight was higher (722.2 g) under no water stress treatment than (104.1 g) under severe water stress. These data demonstrate that fresh fruit wright was six times higher in the no water stress treatment than in the severe water stress treatment. When comparing treatments, this implies that water stress was the greatest contributor to fresh fruit variation. With these inherent fresh fruit weights, it's clear that water stress levels influenced metabolites, resulting in a large fluctuation in African horned cucumber fruit weight. Between no water stress and severe water stress, the fresh fruit weight of African horned cucumber fruit varied by 618 g, which was more than the 208 g reported by Nambi et al.^28^, on English cucumbers. The fact that fresh fruit mean value was higher in the absence of water stress but was lower in the presence of severe water stress could indicate that metabolites involved in fruit formation and growth were crucial in fruit biomass.

Previous study conducted by Weng^4^, found significant differences in metabolites content of fruits harvested from differing growth locations. Asparagine has been reported as one of the most vital metabolites that play a central role such as nitrogen circulation and recycling in plant vegetative organs (Fang, 2011). Perhaps, the fact that asparagine was higher in fruit harvested from protected structures compared to open space could be because of constant gaseous exchange in protected structures, as reported by Ahmad^29^.

They discovered that while the osmotic balance within plant organs, particularly leaves, is maintained, there is continuous gaseous exchange because the stomatal pores are open. Because the stomatal pores were working normally in the no water stress and moderate watering treatments, asparagine concentration increased. Gumi and Reinten et al.^30,31^, took this argument a step further in cucumbers and melons, where they discovered varied asparagine content due to different water concentration in the substrate.

Legwaila et al.^27^ reported that abundance of dopa was prominent in crops grown under greenhouse conditions subjected to acidic soil compared to those of open field conditions. The actual cause for high content of this compound on fruit harvested from open space could be higher fruit load, or competition within individual plants; therefore, dopa is synthesised as a response against the competition. This shows that when a plant has more fruit, this metabolite is altered, as it is linked to suppressing dominance from neighboring fruit, according to Weng^4^. Their studies also showed that when plants have a higher fruit load, the amount of dopa in strawberry fruit increases.

Moing et al.^32^, report that dopa content often shifts immediately when there is limited space due to high fruit load in cucumbers. Other researchers such as Tucker^33^, found that the metabolite dopa was higher in oranges obtained from crops planted at a smaller spacing, but lower in oranges harvested from crops planted at a larger spacing. Similar findings were reported by Hadid^34^ on the other hand, reported that water stress and acidic soil levels were important contributors of this compound's change in apricots, they found that dopa levels in apricot fruit rose when water and acid levels are low. This suggests that fruit dopa levels are affected by water stress and the type of soil used. These data support the current study's conclusion that dopa fluctuation in African horned cucumber fruit is caused by water stress and soil types in different growing environments.

The amount of 4-hydroxyproline in plants exposed to normal watering in protected structures (greenhouse and shade net) increased in this study. Savvides et al.^35^ identified 4-hydroxyproline as a crucial component of plant cells, whereas^19^ noted that 4-hydroxyproline is an important component of the cell membrane because it contains glycoprotein that protects the cell wall. The fact that 4-hydroxyproline levels in fruit increased when water levels were high but reduced when water levels were low suggests that water availability inside plant organs such as xylem and phloem may impact plant cell membranes. The current findings show that variations in 4-hydroxyproline in fruit are strongly related to water availability and the growth environment.

Fang^5^ showed significant variation in metabolites content of crops cultivated under protected structures and subjected to different water stress levels when compared to those that were not water stressed. Excessive stress produced by environmental factors causes plants to evolve defence mechanisms^36^. When compared to the open space environment, 5-glutamylcysteine abundance was higher in protected structures (greenhouse and shade net). This is likely owing to environmental factors in protected structures (greenhouse and shade net), as the crop naturally grows in the wild^25^. The different environmental conditions, such as excessive humidity and excess water, induced biochemical activity such as enzymes, which caused variation in 5-glutamycysteine abundance on fruit.

Regarding acetylcarnitine, the study found a substantial difference between treatment interactions of water stress levels and soil types under different growing conditions during all seasons. Water stress, which subsequently reduced solute movement via xylem and phloem due to stomatal closure, could be the main cause of this compound's variation in fruit. According to^25,37^, this compound serves as a carrier for transporting fatty acids into mitochondria for cell activities, subsequently improving plant health. Tona et al.^26^, discovered that water stress caused significant decrease in acetylcarnitine, but normal watering increases its content in melons.

Friedman^38^, discovered differences in the acetylcarnitine concentration of cucumber fruit grown from plants with different genetic characteristics and water levels. Acetylcarnitine increased greatly when the water level was normal, but it reduced when the water level was low. Due to the application of normal watering and the use of loam soil in the greenhouse environment, acetylcarnitine levels increased dramatically in this study. Perhaps the actual cause for this significant increase in the greenhouse environment could be that the treatments imposed increased evapotranspiration rate, subsequently resulting in improved osmotic pressure, positively affecting dissolved mineral movement within xylem as reported by Dold and Cocks and Tucker^33,39^ on cucumbers.

Kynurenine significantly increased under moderate water stress treatment and loamy soil. Similar findings were reported by Fang^5^, who found that this compound effectively inhibit ethylene in plant root tissue, consequently reducing fruit growth and development. The significant increase in abundance of this compound could be that moderate water stress combined with loamy soil created ideal conditions for anaerobic respiration of plant roots, as it was discovered by Hu et al. and Moco et al.^40,41^, who remarked that lower ethylene causes delay in tuber development.

In this study, norepinephrine content was not significantly affected by interaction of water stress levels and soil types under different growing environments. However, there was significant abundance of this metabolite at open space environment. Hadid and Shu et al.^34,42^ explain the function of this compound as being involved in regulating physiological functions such as carbohydrate metabolism and stress tolerance. Variation in abundance of norepinephrine due to growing conditions could be linked with sunlight intensity and evapotranspiration rate. Under open space environment, norepinephrine significantly increased, but decreased under greenhouse and shade net conditions. Similar findings were observed by Sanchez et al.^43^, who found that stomatal opening and closure caused by radiation has a direct influence on the abundance of norepinephrine in cucumber.

Evidence from the present study reveals that treatment of moderate stress combined with sandy loam under greenhouse environment significantly caused an increase in abundance of niacinamide when compared to other treatments. Since the crop grows naturally in the wild, the variation in niacinamide content could be due to greenhouse temperatures, which negatively affected enzymic activities, stomatal opening and closure, as reported by Flemotomou and Mukherjee et al.^44,45^, who discovered that greenhouse-grown fruit had a higher niacinamide content than open field fruit. The current study's findings revealed that growing environments are the primary cause of this niacinamide volatility, thus affecting its abundance in fruit.

## Conclusions and future research

The study showed that primary metabolites shift of African horned cucumber fruit are indeed affected by different treatment combinations, such as different irrigation water regimes and soil types at varying growing conditions. The key findings of this study are as follows: primary metabolites such as asparagine, dopa, 4-hydroxyproline, 5-glutamylcystiene, acetylcarnitine, kynurenine and niacinamide were higher in protected growth structures when compared to open field environment, but norepinephrine was higher in open field conditions when compared to protected growth structures (greenhouse and shade net) conditions. This great shift in metabolites profile could be a possible explanation of yield variation between protected growth structures (greenhouse and shade net) compared to open field. This is a future alternative crop, therefore, understanding its growth habits, biological/biochemical constituents and metabolites shifts is important for commercialization, consequently, help farmers improve yield and profit maximisation.
